# Supplementary material for: Analysis of Human Milk Microbiota in Northern Greece by Comparative 16S rRNA Sequencing vs. Local Dairy Animals
Source: Nutrients. 2024 Jul 9;16(14):2175. doi: 10.3390/nu16142175 (PMC11280067; doi:10.3390/nu16142175)
Supplement: Supplementary file 1 [file nutrients-16-02175-s001.zip › Supplementary Figures.pdf]

Supplementary Material

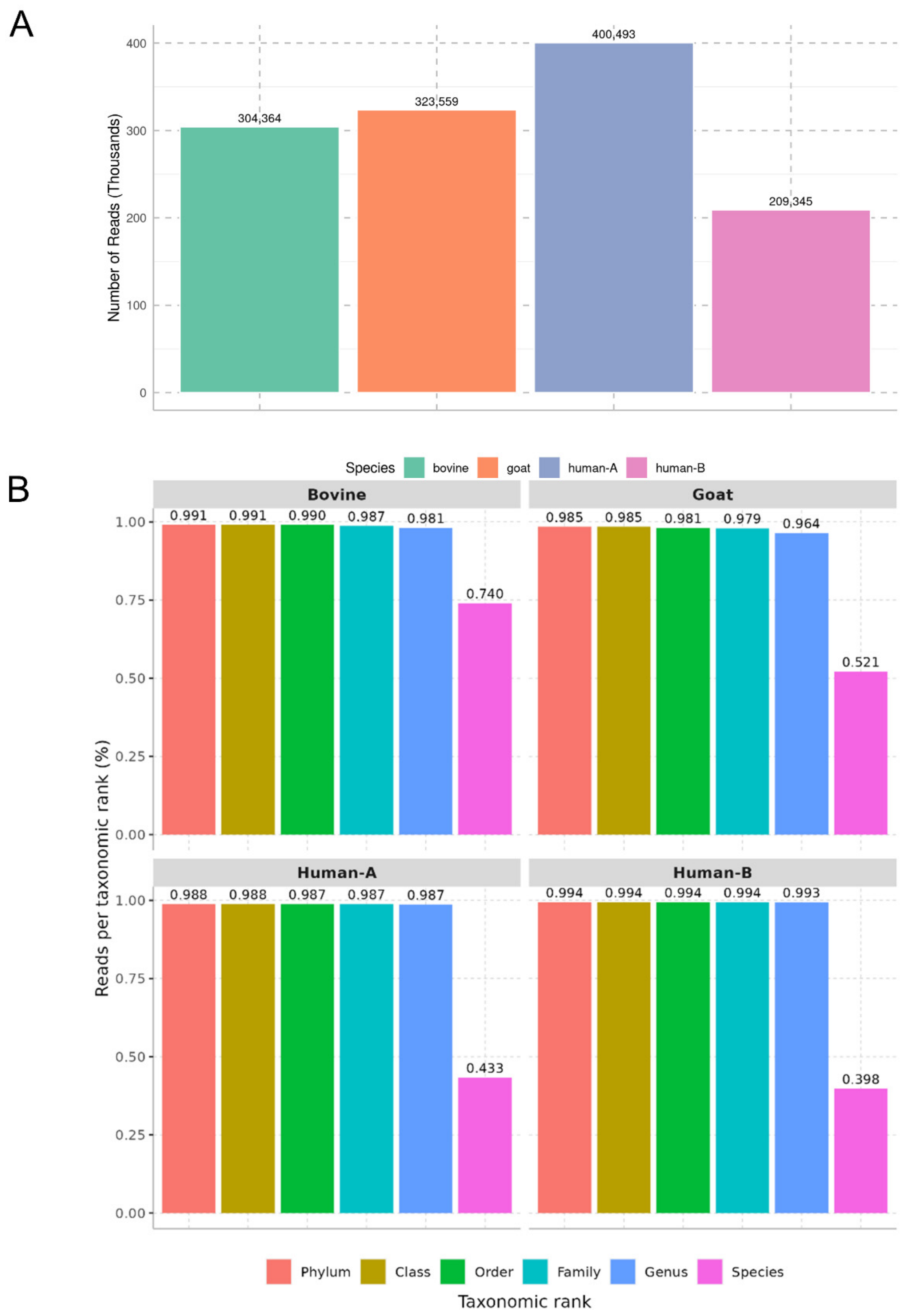

Figure S1. Overview of 16S rRNA sequencing in milk samples from human (A: colostrum milk, B: mature milk), bovine and goat. A. Number of reads across milk samples. B. Relative proportion of the reads assigned in each taxonomic rank

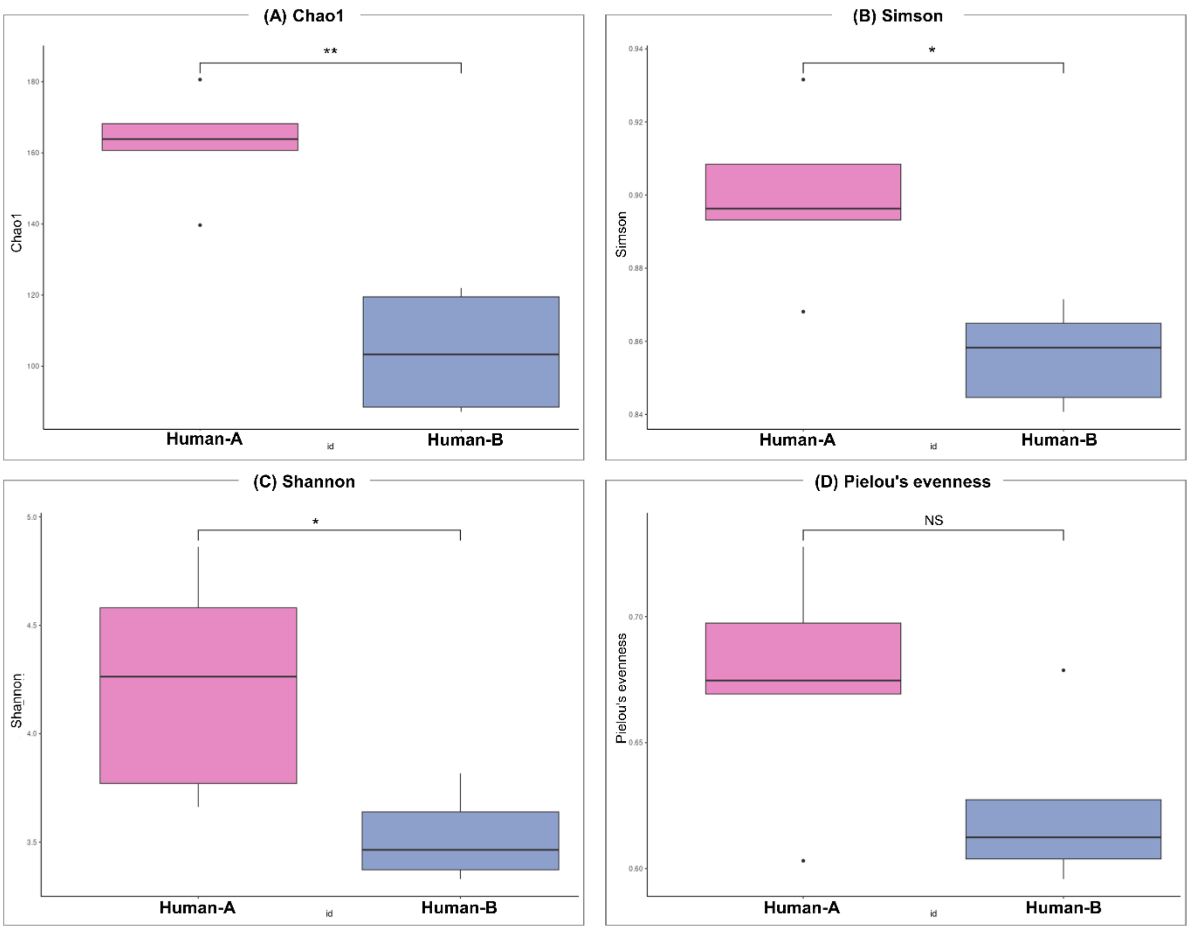

Figure S2. Human colostrum (Human-A) and mature (Human-B) milk microbiome alpha-diversity. (A) Chao1, (B) Simson, (C) Shannon indices and (D) Pielou's evenness. Statistical significance (Wilcoxon test); NS $p > 0.05$, * $p < 0.05$, ** $p < 0.001$. Human-A: Colostrum milk, Human-B: Mature milk.

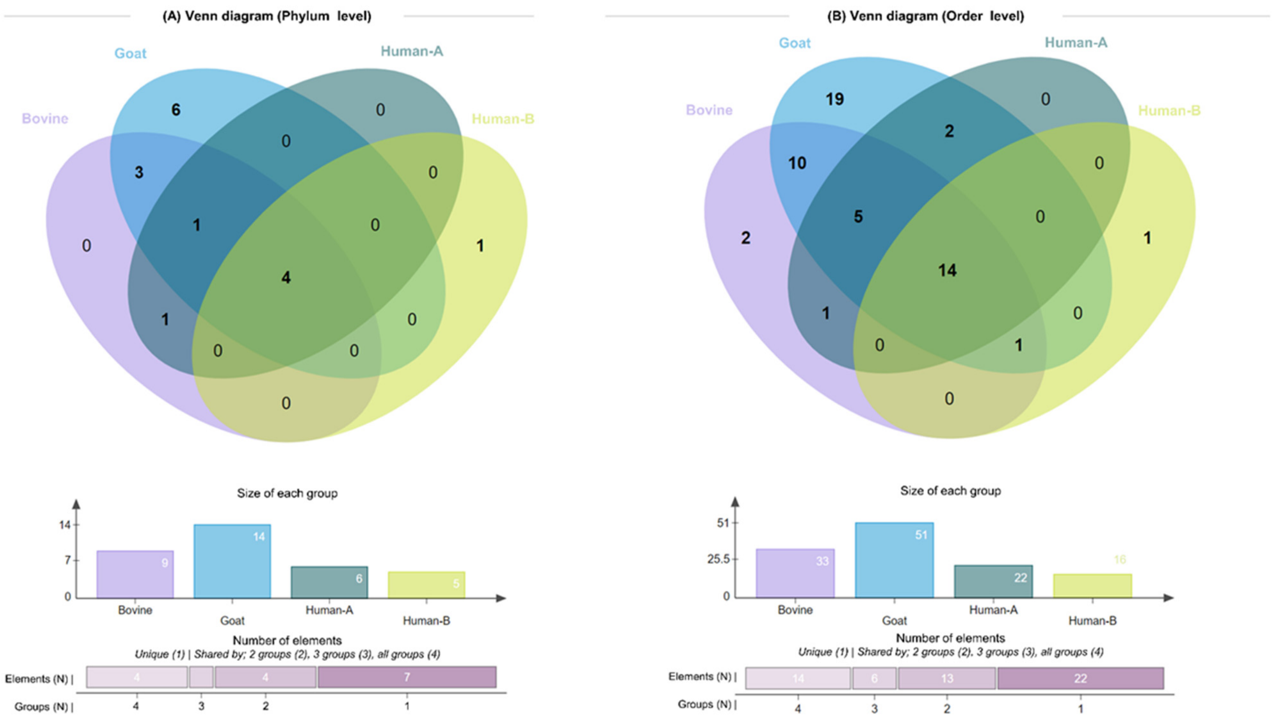

Figure S3. Venn Diagram Illustrating the overlap of bacterial (A) Phyla and (B) Order, across human, bovine and goat milk samples. Amplicon Sequence Variants (ASVs) had to be detected in at least 4 out of 5 samples to ensure robustness in the comparative assessment. Human-A: colostrum milk, Human-B: mature milk.

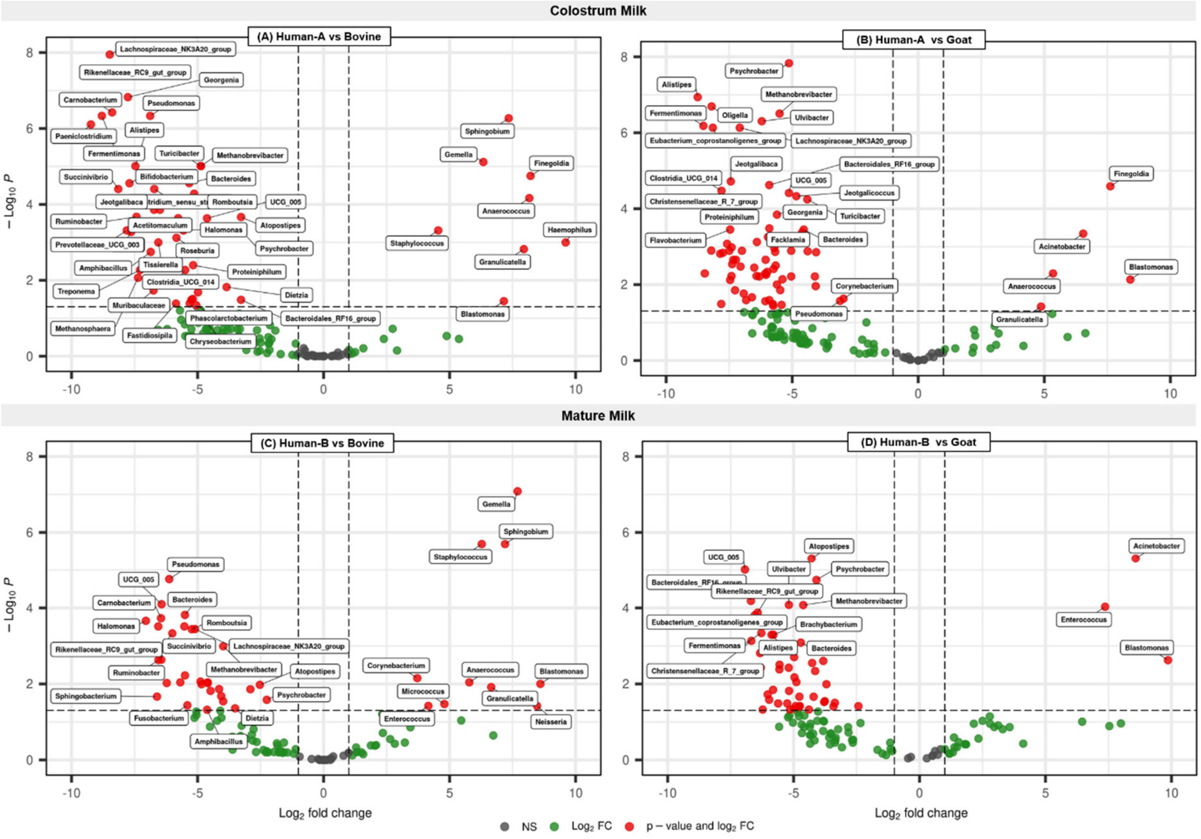

Figure S4. Differential abundance analysis (DAA) in genus level of ASV resolution. For colostrum milk; comparison between (A) human (n=5) and bovine (n=5, control) milk samples, (B) human and goat (n=5, control) mature milk samples. For mature milk; comparison between (A) human (n=5) and bovine (n=5, control) milk samples, (B) human and goat (n=5, control) mature milk samples. Genera with adjusted p-value greater than 0.05 and log_2(Fold change) greater than 1 were considered statistically significant (red color).
